# Catastrophic consequences: can the feline parasite *Toxoplasma gondii* prompt the purrfect neuroinflammatory storm following traumatic brain injury?

**DOI:** 10.1186/s12974-020-01885-3

**Published:** 2020-07-25

**Authors:** Tamara L. Baker, Mujun Sun, Bridgette D. Semple, Shiraz Tyebji, Christopher J. Tonkin, Richelle Mychasiuk, Sandy R. Shultz

**Affiliations:** 1 Department of Neuroscience, Monash University, 6th Floor, The Alfred Centre, 99 Commercial Road, Melbourne, VIC 3004 Australia; 2 Department of Medicine, The University of Melbourne, Parkville, VIC Australia; 3 Division of Infectious Diseases and Defence, The Walter and Eliza Hall Institute of Medical Research, Parkville, VIC Australia

**Keywords:** Parasite, Infection, Neuroinflammation, Immune response, Microglia, Astrocytes

## Abstract

Traumatic brain injury (TBI) is one of the leading causes of morbidity and mortality worldwide; however, treatment development is hindered by the heterogenous nature of TBI presentation and pathophysiology. In particular, the degree of neuroinflammation after TBI varies between individuals and may be modified by other factors such as infection. *Toxoplasma gondii*, a parasite that infects approximately one-third of the world’s population, has a tropism for brain tissue and can persist as a life-long infection. Importantly, there is notable overlap in the pathophysiology between TBI and *T*. *gondii* infection, including neuroinflammation. This paper will review current understandings of the clinical problems, pathophysiological mechanisms, and functional outcomes of TBI and *T*. *gondii*, before considering the potential synergy between the two conditions. In particular, the discussion will focus on neuroinflammatory processes such as microglial activation, inflammatory cytokines, and peripheral immune cell recruitment that occur during *T*. *gondii* infection and after TBI. We will present the notion that these overlapping pathologies in TBI individuals with a chronic *T*. *gondii* infection have the strong potential to exacerbate neuroinflammation and related brain damage, leading to amplified functional deficits. The impact of chronic *T*. *gondii* infection on TBI should therefore be investigated in both preclinical and clinical studies as the possible interplay could influence treatment strategies.

## Background

Traumatic brain injury (TBI) is a leading cause of morbidity and mortality worldwide, yet there have been no successful phase III TBI clinical trials to date [1–3]. To develop effective TBI interventions, it is imperative that we understand the underlying mechanisms that drive negative outcomes, as well as the factors that can modify TBI pathophysiology [4, 5]. For example, neuroinflammation is recognized as a key secondary injury mechanism in TBI, and initial studies indicate that the presence of concurrent immune stressors can alter the aftermath of TBI [6–9]. However, despite the important implications of this phenomenon in understanding TBI heterogeneity and optimizing interventions, it remains an understudied topic [10].

One possible mechanism that can contribute to neuroinflammation is an underlying infection. *Toxoplasma gondii* is a parasite that infects approximately one-third of the world’s population [11]. In other words, many people who sustain a TBI are likely already infected with *T*. *gondii* at the time of injury. *T*. *gondii* has a tropism for brain tissue in intermediate hosts such as rodents and humans, and through the development of cysts, chronic life-long infection ensues [11]. Notably, there is significant overlap between the pathophysiology of *T*. *gondii* and TBI, including the activation of similar neuroinflammatory pathways [12, 13]. Considering the prevalence and pathophysiological similarities of TBI and *T*. *gondii*, it is important to consider the potential impact of *T*. *gondii* infection on TBI outcomes and appropriate intervention strategies. However, to our knowledge, the effect of *T*. *gondii* on TBI outcomes has never been studied. Therefore, this review will highlight the clinical problems, neuroinflammatory pathways, and functional consequences of TBI and *T*. *gondii* separately, before discussing the potential synergistic effects of *T*. *gondii* infection in individuals who have sustained a TBI. We conclude by emphasizing the need for further research into this relationship and provide suggestions for future studies.

## Traumatic brain injury

### Clinical problem of TBI

TBI is a key contributor to the global burden of disease [2, 14]. Reported incidence rates per country markedly vary depending on case definition, and are influenced by a lack of diagnosis, reporting, and medical attention being sought for mild TBI individuals [15–17]. Globally, estimates of annual incidence range from 47.3 to 1322/100,000 depending on region, with most estimates throughout the Western world being placed around 250–350/100,000 [2, 18]. This equates to between 10 and 27 million new cases of TBI each year worldwide; however, this is believed to be an underestimation [15]. Furthermore, since 1990, there has been an increase of 77% in the absolute number of disability-adjusted life-years as a result of TBI [19]. This emphasizes not only the lasting impact of a TBI but also the extent to which this global health burden continues to grow. Additionally, TBI is associated with the development of neurological and mental disorders such as post-traumatic epilepsy (PTE) [20], major depressive disorder [21], and schizophrenia [22], while also being a risk factor for neurodegenerative diseases including Alzheimer’s disease (AD) [23] and Parkinson’s disease [24].

Despite promising pre-clinical and phase II clinical trials in TBI, to date, no phase III clinical trial has identified a therapy that improves TBI recovery [1, 3]. This reflects not only the barrier posed by the heterogenous nature of TBI pathophysiology and presentation but also the juxtaposition of variability seen within preclinical and clinical study designs. In a clinical setting, TBI varies in injury mechanism and severity, as well as pre-injury vulnerabilities such as age, sex, and genetic factors [4]. Pre-injury vulnerabilities, or even the presence of other concurrent factors such as infection, may alter TBI pathophysiology and outcomes [5, 10, 25–27]. On the other hand, preclinical animal models, which ultimately provide the foundation for clinical trials, are highly homogenous as they typically utilize isolated TBI platforms that often fail to incorporate the heterogeneity of the clinical population [4]. This discrepancy demonstrates the necessity to expeditiously study TBI pathophysiology, the clinical factors that modify it, and develop implementable intervention strategies.

### TBI pathophysiology

#### Primary mechanisms

The neurological damage associated with TBI may result from a range of pathophysiological mechanisms. ‘Primary injury’ is the result of direct mechanical forces, most commonly resulting from falls, motor vehicle accidents, assaults, and war zone injuries [2, 18, 28]. These direct forces can lead to the rapid onset of largely irreversible mechanical disruptions to brain tissue. Such disruptions may include direct cell death, torn or stretched axons, and damage to the blood-brain barrier (BBB), all of which are considered to be hallmarks of TBI [29–31].

As a result of the mechanical insult, neurons can sustain damage that leads to ionic flux and inappropriate depolarization [32]. For example, neurons become depolarized resulting in an influx of calcium to the presynaptic cell, causing a large release of the excitatory neurotransmitter glutamate, into the synaptic cleft [33]. This release has been shown through microdialysis studies in both humans and rodents to occur in a force-dependent manner, within minutes of sustaining a TBI [34–36]. Glutamate then acts on α-amino-3-hydroxy-5-methyl-4-isoxazole-propionic acid and *N*-methyl-d-aspartate receptors on the post-synaptic neuron, leading to increased calcium entry [33]. Increased intracellular calcium can further lead to intermediate early gene activation, disruption of mitochondrial production of adenosine triphosphate (ATP), activation of proteases and kinases, and increased production of reactive oxygen species (ROS) [37]. Increased extracellular glutamate can result in decreased expression of glutamate (GLT)-1 or excitatory amino acid transporter-2 transporters on astrocytes [38]. This can cause glutamate to remain in the synaptic cleft resulting in excitotoxicity, which can then lead to further cell death, neuronal injury, and dysfunction of surviving neurons [33].

#### Secondary mechanisms

Within minutes to days after the primary insult, a myriad of secondary pathological pathways begins. These include neuroinflammation, excitotoxicity, oxidative stress, apoptotic cell death, and further BBB disruption, among others [12, 31, 33, 39, 40]. One of the most common and influential processes to occur is neuroinflammation [12, 41].

Damaged and dying cells release cellular debris including alarmins such as ATP, ROS, and high-mobility group box 1 (HMGB1) [12]. Alarmins are recognized by pattern recognition receptors (PRRs) on glial and immune cells, including toll-like receptors (TLRs) and purinergic receptors [42]. For example, microglia, which act as the primary resident immune cells in the central nervous system (CNS), have been demonstrated to be activated via TLRs (e.g., TLR4) in Sprague-Dawley rats following fluid percussion injury [43]. Microglial activation leads to expression of myeloid differentiation primary response protein 88 (MyD88), nuclear factor κ-light-chain-enhancer of activated B cells (NF-κB) activation, and downstream cascades of immune signaling [44, 45]. This includes release of pro-inflammatory cytokines and chemokines such as interleukin (IL)-1β, IL-6, IL-12, tumor necrosis factor-α (TNFα), CC chemokine ligand (CCL)2, CXC chemokine ligand (CXCL)8 and CXCL9, as well as release of oxidative metabolites including nitric oxide (NO) and ROS [12, 46, 47]. Moreover, both alarmins and NF-κB activation have been shown to lead to increased expression of the nucleotide-binding oligomerization domain-like receptor family pyrin domain-containing 3 (NLRP3) inflammasome in rodents, causing secretion of the pro-inflammatory cytokines IL-1β and IL-18 [48, 49]. As the released inflammatory mediators bind receptors on ‘surveillant’ microglia and trigger their activation, a self-propagating cycle begins, leading to tissue damage, neurotoxic effects, and dysregulated microglial activation [46, 50–52]. Furthermore, both experimental and clinical studies have demonstrated that microglia activation can persist for months to years depending on the severity and recurrence of TBI, meaning that tissue repair can be chronically hindered [53–58]. It should be noted that, depending on phenotype, microglia can also aid in phagocytosis and remyelination, as well as in the release of anti-inflammatory mediators such as IL-10, transforming growth factor-β and insulin-like growth factor-1; all of which provide benefit following TBI [50, 59].

Astrocytes are also important in neuroinflammation. Following TBI, astrocytes respond via reactive astrogliosis, a transformation that includes gene expression changes and cell hypertrophy to establish a glial scar; the latter of which is thought to be beneficial by isolating the damaged tissue and preventing further cell loss [60, 61]. Similar to microglia, PRRs on astrocytes can be activated by alarmins such as HMGB1 [62]. For example, activation of TLR4 can signal MyD88-dependent and -independent pathways, and NF-κB activation [63]. Downstream release of IL-1β, IL-6, TNF-α, CCL5, CXCL1, CXCL2, CXCL10, granulocyte-macrophage colony-stimulating factor (GM-CSF), and NO may henceforth occur as demonstrated by both preclinical and clinical studies [60, 64–66]. These inflammatory mediators coupled with those released by microglia, such as IL-1β, stimulate further cytokine secretion through NF-κB-dependent mechanisms, and may therefore play a role in excitotoxicity [67]. However, the level of reactive astrogliosis is heterogenous and depends on the nature and severity of the TBI [68].

Pro-inflammatory mediators can contribute to BBB permeability and breakdown, and facilitate the infiltration of peripheral immune cells [31, 69, 70]. For example, IL-1β, CXCL8 and CCL2 are key mediators in neutrophil and monocyte/macrophage migration into the injured brain parenchyma [71]. As such, it has been demonstrated with a controlled cortical impact (CCI) that within 24 h of the primary injury, levels of circulating neutrophils increase, and these cells are the first wave of peripheral immune cells to be found within the injury site [72]. Once in the brain parenchyma, neutrophils can become activated by inflammatory mediators and chemokines such as IL-1β, TNFα, CXCL1, CXCL2, and CXCL8 [73]. Alternatively, neutrophils may interact and become activated via cell-cell contact with astrocytes, leading to increased expression of IL-1β, IL-6, TNFα, and CXCL2 [74]. Neutrophils additionally release ROS which contributes to secondary injury [75, 76], and can reciprocally activate microglia, causing a synergistic activation cascade [73]. After this initial wave, murine studies have shown that macrophages become the predominant infiltrating leukocyte and contribute to the production of inflammatory cytokines such as TNFα upon activation [77–79]. Macrophages and other monocytes do, however, also have beneficial neurological properties, such as removal of debris through phagocytosis and assisting remyelination [59].

T cell infiltration into the injury site is facilitated by BBB disruption and regulatory T cell depletion [79–81]. Once at the injury site, T cells have been shown to become activated to produce T-helper 1 cytokines, such as TNFα and IFNγ, in mice following CCI [82]. Furthermore, the release of IFNγ signals the upregulation of major histocompatibility complex class II protein in microglia (priming the microglia), the release of chemokines and oxidative metabolites, and the further release of pro-inflammatory cytokines [83–85]. However, it has been demonstrated that T lymphopenia occurs after CCI in C57BL/6 mice, potentially as a result of injury-related thymic atrophy and deficits in T cell maturation [79]. These changes may be coupled with alterations in other immune cell functions as well as lymphoid organs, thereby characterizing peripheral immunosuppression in the acute, sub-acute and even chronic stages post-injury [79, 86].

### Functional consequences of TBI

Following a TBI, cognitive, motor, and emotional abnormalities have been demonstrated in both human and murine studies; however, depending on the nature of the TBI, these consequences may be transient or long-lasting [87–91]. Neuroinflammation likely contributes to these deficits, although more research is required to determine the precise mechanisms [92–94]. For example, NLRP3 inflammasome activation has been associated with poor spatial memory in rodents, and elevated serum levels of IL-1β and IL-18 are correlated with increased cognitive impairment in humans [48, 95, 96]. TBI and the later development of neurofibrillary tangles and amyloid-β plaque deposition has also been implicated in the presentation of cognitive deficits in both pre-clinical and clinical studies [97–100]. The mechanisms underlying these cognitive impairments are not fully understood; however, synaptic dysfunction as a result of the amyloid-β and tau may be a mechanistic link [101]. Post-injury neuroinflammation has also been linked to motor deficits through association with peripheral cell recruitment including T cell infiltration and microglia activation in murine models [80, 102]. In addition, prolonged anxiety-like behavior as a result of TBI has been reported in both rodents and humans [93, 103–105]. Moreover, increases in anxiety-like behavior have been associated with imbalances of gamma-amino butyric acid (GABA) and a loss of GABAergic interneurons in the basolateral amygdala [106].

PTE is linked to neuroinflammation and evidence suggests it may be the underlying mechanism of seizures and epilepsy development [20, 107]. For example, murine studies have demonstrated that IL-6, TNFα, and HMGB1 have pro-ictogenic effects, and potentially act through glutamatergic pathways to promote neuronal hyperexcitability [107–111]. In addition, Il-1β may modulate neuronal hyperexcitability through calcium, glutamatergic, and GABAergic pathways, and the effect of IL-1β on BBB breakdown and neutrophil recruitment may promote epileptogenesis [112, 113]. TLR4 may also play a role in seizure development as TLR4 mutant mice have demonstrated seizure resistance [111].

## *Toxoplasma gondii*

### Clinical problem of *T*. *gondii*

Given that the immune system has evolved to combat pathogens, it is unsurprising that infection, especially in neuronal tissue, could affect TBI outcomes. The direct interaction between infection and TBI would most likely occur in response to common human infections that act chronically and have a tropism for brain tissue. *T*. *gondii* is the perfect pathogen in this regard. First discovered in 1908, *T*. *gondii* is a globally distributed single-celled intracellular parasite that can infect most vertebrates, including humans [114]. Infection often occurs via contaminated food and water, first leading to an acute infection that spreads around the body [11]. *T*. *gondii* then differentiates into a slow growing encysting stage that cannot be cleared by the immune system and resides in a latent form in muscle and the CNS [11]. Prevalence of *T*. *gondii* infection (based on immunoglobulin G antibodies) considerably varies between age groups and geographical regions [115, 116]. Children often present with lower rates of infection; however, estimates of seroprevalence in adults range between 5 and 25% in North America, Western Europe, and East Asia, and upwards of 20% elsewhere [115–126]. Moreover, countries such as Brazil and Indonesia typically reported adult seroprevalence above 60% [115, 124, 127]. Historically, *T*. *gondii* infections have been of great focus in pregnant and immunocompromised populations due to the risk of congenital toxoplasmosis and toxoplasmic encephalitis, respectively [114]. Congenital toxoplasmosis occurs secondarily to maternal infection during or just prior to pregnancy, and can result in a stillborn child, miscarriage, or potential visual or cognitive impairments for the child [128]. Furthermore, in immunocompromised individuals, toxoplasmic encephalitis can result from a lack of lymphocyte recruitment to be able to control parasite replication [129]. This condition is characterized by severe neurological symptoms including seizures, edema, and necrosis of brain tissue [130–132]. In immunocompetent individuals, the infection is typically latent and presents asymptomatically [114]. However, it should be noted that, although immunocompetent individuals appear asymptomatic, chronic infection has been associated with subtle cognitive and behavioral changes [133]. These observations have therefore led to a growing field of research in recent years investigating the potential relationship between *T*. *gondii* infection and neurological disorders [134, 135].

To date, no cure exists for *T*. *gondii* infections [136, 137]. This reflects not only the ability of *T*. *gondii* to evade the immune system and establish a latent infection but also the heterogeneity of *T*. *gondii* strains in the clinical population [138]. Strains noticeably differ in virulence, clinical outcomes, and prevalence between regions [139, 140]. In North America and Europe, approximately 90% of reported samples can be classified into one of three intercontinental clonal lineages (types I, II, and III) which exhibit approximately 1–2% differences at the nucleotide level [141, 142]. Although these lineages are prevalent worldwide [142–144], regional clonal lineages such as Africa 1 and Chinese 1 are also common in Africa and Asia, respectively [145, 146]. Moreover, a great deal of diversity has arisen in South America as a result of atypical and recombinant strains, and as such clonal lineage prevalence is low [140]. In humans, type II strains (e.g., typified by the isolates Prugniaud and ME49) are strongly associated with infection irrespective of clinical presentation; however, differences in virulence have been suggested due to associations of type I and type III strains with cerebral and pulmonary toxoplasmosis, respectively [139]. The vast diversity between strains exemplifies the need to study *T*. *gondii* virulence effectors, how clinical outcomes vary between strains, and increase awareness of prevention strategies in regions with high *T*. *gondii* seroprevalence.

### *T*. *gondii* pathophysiology

#### Acute stage

**Fig. 1:**
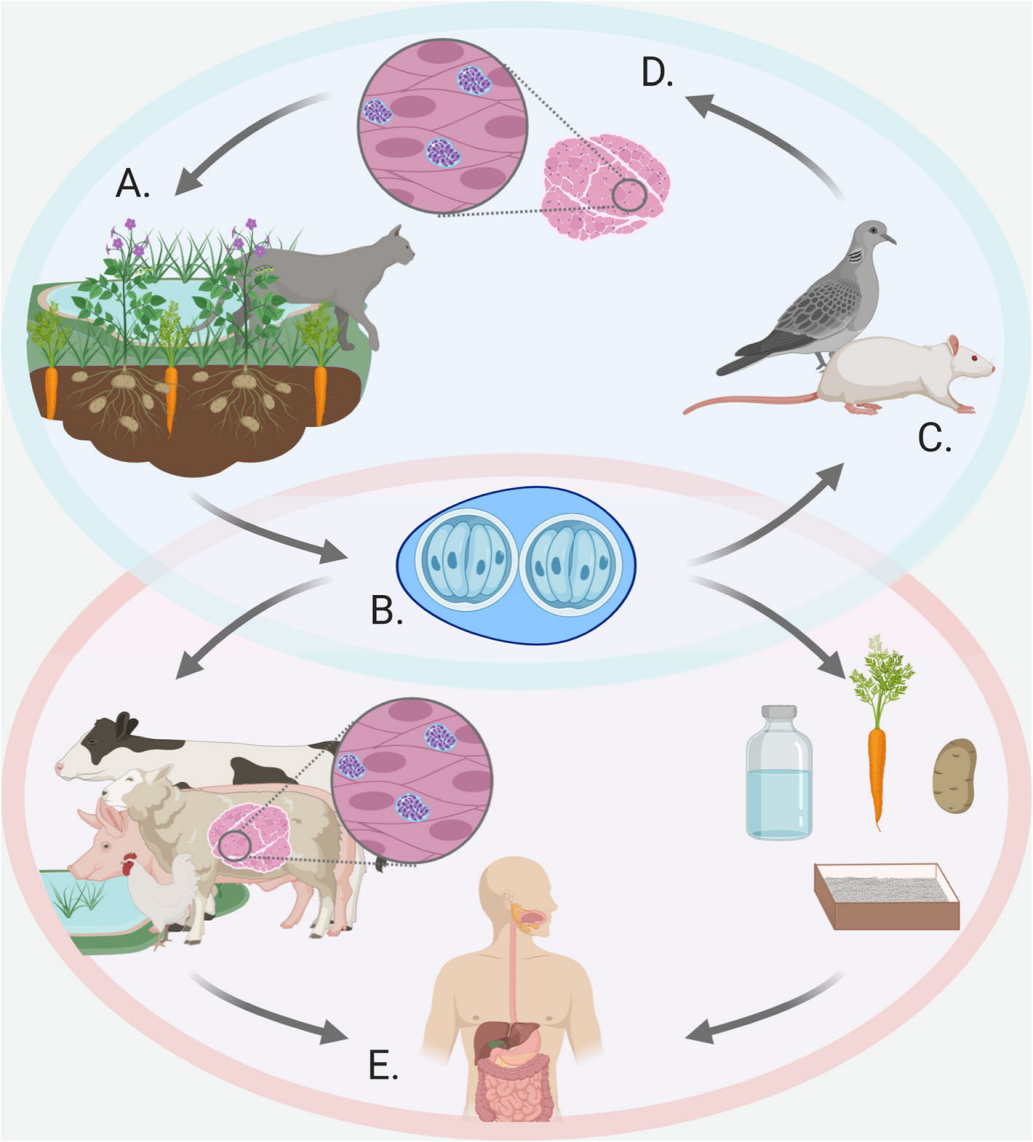
Life cycle of *T*. *gondii* and routes to the human. **a***T*. *gondii* sexually reproduces in cats where oocysts are shed in feces. **b**, **c** Once sporulated, the oocysts become infectious and can be transmitted to rodents or birds via contaminated food or water sources. **d** In these hosts, *T*. *gondii* bradyzoites develop in the brain, skeletal muscle and heart, among other tissues, which are transmitted back to the cat if consumed. **e** Infection of a human can occur via consumption of meat containing bradyzoites or contaminated food, water, or contact with cat litter. Figure created with BioRender.com

*T*. *gondii* is an obligate intracellular parasite, meaning that it must invade host cells to survive and proliferate [11]. To move through tissue, *T. gondii* uses a unique form of cellular locomotion called ‘gliding motility,’ where it then recognizes cognate host cells and initiates a molecular program to invade [147]. Upon invasion, *T. gondii* creates a vacuole around itself (i.e., a parasitophorous vacuole), thereby separating itself from the host cell cytoplasm [147]. *T*. *gondii* has a complex life cycle whereby sexual reproduction can only take place in Felidae family members such as domestic cats (Fig. 1) [11]. From Felidae members, encysted forms of the parasite known as oocysts are shed in fecal matter and can persist in soil for more than a year [11, 148]. Furthermore, oocysts are infectious to a wide variety of vertebrates, including humans, and are transmitted when consuming contaminated food and water sources [11, 114]. Upon ingestion, oocysts release sporozoite forms which infect intestinal tissue, differentiate into acute tachyzoite forms, and then spread throughout the body [11].. *T*. *gondii* can therefore continue a pattern of invasion, replication and egress within a range of cells, causing tissue damage and toxoplasmosis [147]. Clinical symptoms of acute infection often present as mild flu-like symptoms in people with mature and healthy immune systems, but always result in a chronic infection [149]. The beginning of this chronic stage is characterized by the differentiation of tachyzoites into slower growing bradyzoite forms which develop a glycoprotein-rich wall around the vacuole boundary while living within cells of muscles, organs, and the CNS [150]. Infected vertebrates (e.g., rodents and birds) may therefore contribute to the transmission back to Felidae members; however, incidental infection, such as in humans and livestock, commonly occurs.

*T*. *gondii* is a highly successful parasite due to its ability to manipulate host cells, skew the immune system, suppress clearance, and even cause the hypermigration of monocytes which can permit cellular dissemination from the site of infection to the whole body [151–155]. *T*. *gondii* does this by secreting parasite proteins into the host cell cytoplasm and nucleus, which interfere with several cellular pathways [156–160]. Despite the efforts of the immune system to defend the host, *T*. *gondii* migrates to the relatively immune-privileged CNS. To achieve this, it has been proposed that *T*. *gondii* crosses the BBB via one, or a combination of, three mechanisms; hijacking monocytes in a Trojan horse-like manner, transcellular migration, and paracellular migration [161–164]. Once in the brain parenchyma, murine models have revealed that several TLRs recognize *T*. *gondii* [165]. TLR2 and TLR4 have been linked to glycosylphosphatidylinositol detection and TLR2 in particular is considered essential for host resistance [166, 167]. Moreover, some evidence suggests that TLR2 regulates TNFα and CCL2 production by macrophages and neutrophils, respectively [167, 168]. TLR11 is a key *T*. *gondii* sensor in rodents, and is able to recognize *T*. *gondii* profilin-like protein within endolysosomes, thereby leading to the recruitment of MyD88 and downstream initiation of immune signaling cascades [44, 169, 170]. However, it is important to note that *TLR11* is a non-functional gene in humans, and as such the mechanisms of *T*. *gondii* detection by the human immune system are not fully understood [165]. Nonetheless, in murine models, MyD88 recruitment has been demonstrated to permit production of IL-12 which aids host defence [171]. IL-12 is predominantly produced by non-infected dendritic cells (DCs) during *T*. *gondii* infection [172], thereby promoting T-helper 1 responses and subsequent upregulation of IL-2, IL-10, TNFα, and IFNγ [173].

IFNγ is a major effector molecule that acts as a key regulator in host resistance against *T*. *gondii*, and to date, at least three IFNγ-mediated protective mechanisms have been identified [165, 174]. IFNγ enhances the ability of human macrophages to kill *T*. *gondii* [175]. IFNγ also suppresses the growth of *T*. *gondii* via degradation of tryptophan, through activation of indoleamine 2,3-dioxygenase which converts tryptophan into N-formylkyurenine [176, 177]. As *T*. *gondii* is a tryptophan auxotroph, cell starvation occurs. IFNγ also leads to free radical production and oxidative stress as a result [165].

The invasion of *T*. *gondii* in the brain additionally leads to rapid expression of chemoattractants such as CCL5, CXCL9, and CXCL10, by astrocytes and microglia [178]. These chemoattractants recruit CD8^+^ and CD4^+^ T cells that produce IFNγ [179, 180]. Astrocytic expression of IL-1β, IL-6, TNFα, and GM-CSF is also increased upon *T*. *gondii* infection of the CNS [181]. Upregulation of numerous other inflammatory cytokines including IL-2, IL-4, IL-10, and IL-12 have been noted through rodent studies in the CNS during acute *T*. *gondii* infection [182].

#### Chronic stage

Once in the CNS, tachyzoites differentiate into encysted bradyzoites which are resistant to immune clearance, resulting in life-long chronic infection [183]. These cysts are predominantly found intracellularly in the grey matter, with *T*. *gondii* residing in cell processes [184, 185]. Like in tachyzoite stages, it is highly likely that bradyzoites also manipulate their host cell, although this is far less well understood [186, 187]. One remarkable aspect during infection of the brain is that *T*. *gondii* seems to also ‘inject’ parasite proteins into neurons that it does not infect [184, 185]. While it is not understood what role this has during infection, one possible result of this could be a more widespread immunological modulation of tissue, which could have significant consequences.

In the chronic stage of *T*. *gondii* infection, it has been demonstrated that microglia populations remain in a more ‘pro-inflammatory’ state [188]. This is coupled with levels of numerous inflammatory mediators, such as IL-6, TNFα, IFNγ, and GM-CSF remaining above control levels despite showing a decrease from the acute stage [188]. Furthermore, markers of T cell differentiation and exhaustion are simultaneously expressed, which indicates that IFNγ-mediated inflammatory responses are chronically limited to potentially assist parasite survival [188].

In addition to an inflammatory response, *T*. *gondii* infection causes several neurochemical changes including glutamate and dopamine dysregulation [189, 190]. During chronic infection with *T*. *gondii*, the astrocytic glutamate transporter GLT-1 is reportedly reduced in the forebrain over time, leading to increases in extracellular glutamate and excitotoxicity [189]. Interestingly, the *T*. *gondii* genome contains aromatic amino acid hydroxylase (AAH) genes (AAH1 and AAH2) that encode the levodopa protein (precursor to dopamine), suggesting direct modulation by the parasite [191]. However, the impact of *T*. *gondii* on global dopamine levels remains controversial, with both increases and decreases reported [192–196].

Ultimately, *T*. *gondii* infection leads to alterations in brain structure and function [197]. Of importance, somatosensory regions have been noted to contain lesions, and loss of fiber coherence and density [198]. Reductions in dendritic complexity and length have also been observed, as well as a loss of synaptic complexity [189, 196, 198]. Chronic *T*. *gondii* infection has further been linked to microcirculatory dysfunction and reduced angiogenesis in the brain [199], which may contribute to neurodegenerative processes.

### Functional consequences of *T*. *gondii* infection

As it has been shown and now well accepted that *T*. *gondii* influences the mammalian brain and is associated with some brain disorders [134], to appreciate whether *T*. *gondii* could contribute to TBI, it is important that we review what is already known about the effect this parasite has on the brain. In 1979, it was discovered that *T*. *gondii* infection in rodents caused changes in learning [134]. Since then, murine models have provided insight into the multitude of behavioral alterations that may occur in response to this infection [200]. These include changes in memory and learning, locomotion, anxiety, depression-like behaviors, social behavior, and predator aversion [134]. However, in humans, studies into *T*. *gondii* infection-based changes are limited to epidemiological and serological studies, and as such, are primarily retrospective and associative [135, 201].

Memory and learning changes have received little attention in the literature compared to other behavioral modalities, and the murine studies that have been conducted report inconsistent results [202, 203]. These inconsistencies may be attributed to differences in parasite strain, cyst burden, behavioral paradigm, rodent species, sex, and post-infection time of testing [204–207]. For example, it has been demonstrated that female BALB/c mice infected with the ME49 strain of *T*. *gondii*, but not the Prugniaud strain, exhibit impaired spatial working memory at 2 months post-infection [205]. Overall though, the limited literature points toward spatial learning, working memory, and short-term memory being impaired in murine models due to *T*. *gondii* infection [203–205, 208].

Similarly, there is mixed literature regarding changes in anxiety and depression associated with *T*. *gondii* infection and controversy exists as a result. Overall, most published literature indicates that *T*. *gondii* infected rodents have increased anxiety in both the acute and chronic stages of infection [209–212]. One study, however, found that infected rats displayed decreased anxiety [213]. Associative clinical studies have linked *T*. *gondii* infection to depression [214, 215]; however, only some studies have shown *T*. *gondii* infection to induce depression-like behavior in rodents [210, 216]. Moreover, Bay-Richter and Petersen [212] suggest that depressive-like behavior is only affected in genetically vulnerable *T*. *gondii* rodents. Taken together, post-infection time of testing and gene-environment interactions are important factors that influence the response to infection, and these should be considered in study design and analysis [212].

Social behavior and the link between *T*. *gondii* and neuropsychiatric disorders have been of great interest over the last several years. In particular, the association between chronic *T*. *gondii* infection and schizophrenia has been growing in the literature [217, 218]. Sociability with novel mice is impaired in mice chronically infected with *T*. *gondii*, which may reflect this potential link between *T*. *gondii* and schizophrenia [211].

There is also mixed evidence suggesting an association between chronic *T*. *gondii* infection and neurological conditions such as epilepsy and AD. A previous comprehensive systems analysis found that *T*. *gondii* infection modified pathways involved in epilepsy and neurodegenerative diseases [219], and a recent meta-analysis concluded that *T*. *gondii* infection is a risk factor for AD [220]. There is also preclinical evidence suggesting that *T*. *gondii* infection promotes the development of epilepsy and AD in rodents [208, 221, 222]. On the other hand, a case-controlled study involving 99 epilepsy patients and 99 patients without epilepsy found no association between *T*. *gondii* infection and epilepsy [223]. It has been noted that both hyperphosphorylated tau as well as amyloid-β plaques (i.e., pathologies implicated in epilepsy and AD [224]) can be induced in murine models following chronic *T*. *gondii* infection [221]. In contrast, phagocytosis of amyloid-β has also been demonstrated to be enhanced in chronic *T*. *gondii* infection, indicating a modulatory role of recruited immune cells [225]. These differing conclusions may again be due to differences in the murine models and human studies, as well as the post-infection time of analysis. However, these conflicting results highlight the need to expand the field of *T*. *gondii* research, as well as implement multiple models or endpoints into study designs.

## The pathophysiological links between TBI and *T*. *gondii*

**Fig. 2:**
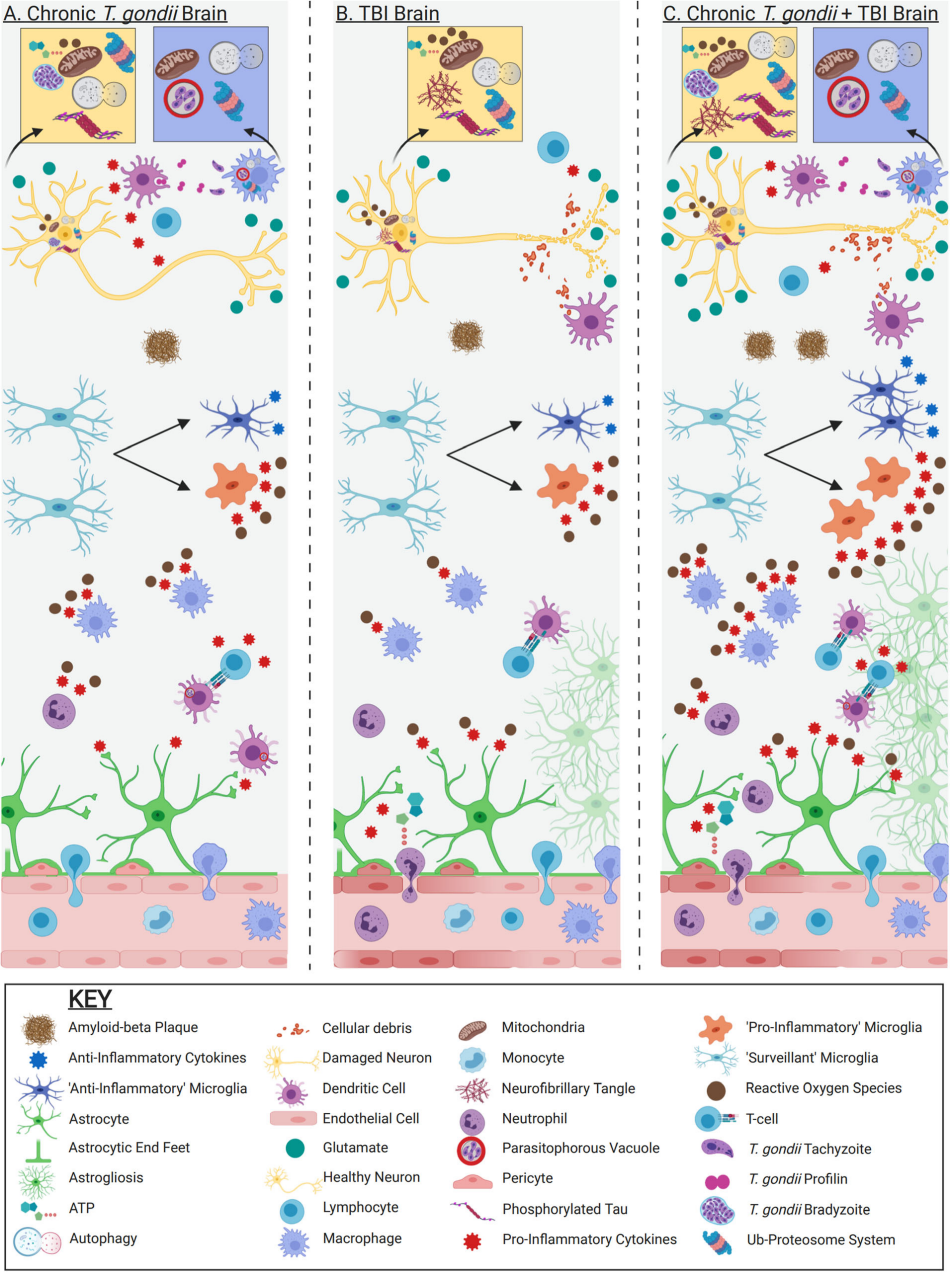
Neuroimmunological processes of chronic *T*. *gondii* infection and TBI, including the hypothesized synergy between conditions. **a** Chronic *T*. *gondii* brain. After migration into brain parenchyma, *T*. *gondii* profilin is detected by non-infected dendritic cells. This leads to production of IL-12 which activates lymphocytes to secrete IFNγ mediating host resistance. Infected and IFNγ-primed dendritic cells prime T cells and trigger production of IFNγ. IFNγ then activates astrocytes, leading to secretion of pro-inflammatory cytokines such as IL-1β, IL-6, and GM-CSF. GM-CSF can prime microglia, leading to the production of TNF-α, IL-6, and ROS. IFNγ and TNFα further activate macrophages, leading to secretion of pro-inflammatory cytokines and ROS, thereby further inhibiting *T*. *gondii* replication in macrophages. However, *T*. *gondii* preferentially infects neurons, and once inside, bradyzoites develop to avoid clearance. **b** TBI. Immediately following injury, damaged pericytes and parenchyma release alarmins such as ATP and ROS. These signaling molecules activate microglia and astrocytes to promote release of inflammatory cytokines and ROS. Leukocyte recruitment to the injury site begins with neutrophil infiltration, followed by macrophages and T cells. In response to cellular debris, T cells and macrophages produce additional pro-inflammatory cytokines. **c** Chronic *T. gondii* + TBI Brain. If an individual harboring a chronic *T*. *gondii* infection were to sustain a TBI, the neuroinflammatory profile may be exacerbated. A greater population of ‘pro-inflammatory’ and ‘anti-inflammatory’ microglia, as well as activated astrocytes, may be present not only at the onset of injury but also post-TBI. Increased populations of these cells may result in an increase of the relative abundance of inflammatory mediators post-TBI. Increased numbers of activated neutrophils, T cell, and macrophages may additionally be present, with the potential for these cells to further produce inflammatory mediators. Additionally, hyperphosphorylated tau and amyloid-β may accumulate more readily, potentially accelerating the neurodegenerative process. Figure created with BioRender.com

Given the overlapping neuroinflammatory processes of TBI and *T*. *gondii* and the known effects that chronic infection can have on the function of the brain (Fig. 2), it is possible that an individual who sustains a TBI while chronically infected with *T*. *gondii* will present with exacerbated neuroinflammation. Also, the large indiscriminate release of glutamate after TBI, coupled with the increased extracellular glutamate in chronic *T*. *gondii* infection, could result in further excitotoxicity and downstream inflammation and phosphorylation of tau [189, 226, 227].

Subclinical neuroinflammation that occurs in a chronic *T*. *gondii* infection could be exacerbated by a TBI due to an increased population of activated microglia and astrocytes. Similar to increases in astrogliosis and activated microglial populations following repeated mild TBIs [228], it is possible a TBI sustained during a chronic *T*. *gondii* infection may act as a ‘second hit’ given that *T*. *gondii* can elicit a ‘pro-inflammatory’ microglial population [188]. In other words, after the insult, primed microglia could excessively produce pro-inflammatory cytokines such as IL-1β, IL-6, IL-12, TNFα, and IFNγ [229], and additional ‘surveillant’ microglia and astrocytes may become activated to produce additional inflammatory mediators. These cascades would result in a greater proportion of astrogliosis and activated microglia compared to TBI individuals without *T*. *gondii*. Moreover, the proposed excessive inflammatory mediators secreted acutely after TBI may activate the sympathetic nervous system (SNS) and hypothalamic-pituitary-adrenal (HPA) axis, resulting in an exacerbated systemic inflammatory response [230, 231]. Negative feedback via the SNS and HPA axis could therefore be triggered, leading to increased peripheral immune cell and lymphoid organ dysfunction, and more pronounced immunosuppression compared to TBI individuals without infection [232]. As immunosuppression can result from TBI [79], it is also plausible that individuals may exhibit symptoms of toxoplasmic encephalitis given that control of chronic *T*. *gondii* infection requires continual immune cell infiltration [129, 233]. In other words, individuals may be vulnerable to tachyzoite proliferation and subsequent increased neuroinflammatory sequelae sub-acutely or chronically post-injury. An individual may, for example, present with increased necrotic cell death and lesional volume due to increased inflammatory-related cell damage. This could have further long-term consequences of exacerbated functional deficits.

With regards to glutamate and excitotoxicity, murine studies have also suggested a *T*. *gondii*-induced reduction of astrocytic GLT-1, which is likely to exacerbate TBI-induced excitotoxic effects if a chronic infection were to precede a TBI [33, 189]. This could further dysregulate GLT-1 post-TBI, thereby contributing to increased excitotoxicity and neuronal damage or death [234]. The temporal profile of elevated glutamate may additionally be altered if the insults occur together, given that in humans, severe TBI alone can result in elevated glutamate that can persist for days or weeks in some brain structures [235, 236].

The changes to the brain’s structure and function, including alterations to dendrites, synapses, and microvasculature as a result of chronic *T*. *gondii* [197], together with the widespread injury to axons, neurons, and cerebrovasculature induced by the mechanical insult of the TBI may cause an increased lesion volume if the conditions occur concurrently [237]. The increased level of ROS, NO, and oxidative stress as a result of TBI, compounded with increased NO in chronic *T*. *gondii* infection, may additionally trigger profuse neuronal death [238, 239]. As ROS facilitates BBB permeability and breakdown [69, 240], excessive ROS in combination with increased pro-inflammatory cytokines could escalate BBB breakdown and peripheral cell recruitment, dysregulate ionic flux across the barrier, and increase lesion volume [240, 241]. Furthermore, as amyloid-β accumulation and hyperphosphorylated tau have been implicated in both *T*. *gondii* and TBI separately [221, 242], when the conditions occur simultaneously, an amplification of these products could occur, further exacerbating oxidative stress and associated damage, and accelerating neurodegenerative changes [243, 244].

In terms of behavioral outcomes, as mentioned previously, *T*. *gondii* infection alone has been shown to induce subtle deficits in cognitive functioning, motor functioning, and social behavior [134, 211]. Adding to this, *T*. *gondii* infection has been associated with increased anxiety and depression [211, 212]. Coupled with a TBI, which is causal to similar impairments, an exacerbation of deficits in functional outcomes could occur. For example, given the links between *T*. *gondii* and TBI with epilepsy [20, 245], it is possible that epileptogenesis could occur more readily as a result of exacerbated IL-1β production, glutamatergic and GABAergic pathways. Moreover, *T*. *gondii* infection could lead to temporal changes in deficits that occur with TBI, such that deficits occur over a long-term scale rather than transiently. This would mean worse functional outcomes after TBI in individuals chronically infected with *T*. *gondii* compared to those without infection, and supplementary rehabilitation may be necessary. Additionally, the possible cumulative effect may result in lifelong functional deficits as a result of increased structural damage. Together, *T*. *gondii* and TBI may also accelerate neurodegeneration given that both are independently implicated in the production of neuroinflammation, hyperphosphorylated tau and amyloid-β plaques, as well as alterations to glutamatergic and GABAergic pathways [106, 189, 221, 242]. Therefore, individuals may be at an increased risk of developing chronic neurological conditions such as PTE and AD, among others [20, 23, 208].

The potential for synergism between *T*. *gondii* and TBI is further complicated by factors such as biological sex and age, as both are known to modify neuroinflammatory and recovery pathways. For example, murine studies have demonstrated a divergent response up to 7 days post-injury, with more robust microglial activation, astrogliosis, and cell loss in young adult males compared to young adult females after a moderate to severe CCI [246, 247]. Therefore, when coupled with a chronic *T*. *gondii* infection, young adult males that have sustained a TBI may have worse outcomes compared to their female counterparts. Though, it should be noted that although preclinical studies often report better outcomes in females, the findings are mixed in human studies [248, 249]. Regarding the impact of age, preclinical studies show that aged rodents have exacerbated microglial activation and pro-inflammatory cytokines coupled with increased functional deficits post-injury compared to young-adult rodents [250–252]. Given that *T*. *gondii* seroprevalence increases with age, elderly individuals may be at an increased risk of experiencing the two conditions concurrently and may experience worse outcomes post-injury compared to younger TBI individuals with chronic *T*. *gondii* infection [120].

## Future directions

This review has highlighted numerous areas of pathological overlap between *T*. *gondii* and TBI, which in theory could exacerbate the functional consequences of these conditions. With that said, to the best of our knowledge, no literature examining the overlap between *T*. *gondii* and TBI exists, and a great deal of future research is still required. First and foremost, studies must be done to comprehensively characterize if and how *T*. *gondii* infection modifies the aftermath of TBI. Animal models will be invaluable to assist in characterizing the hypothesized synergism between *T*. *gondii* and TBI, and validated rodent models of TBI and *T*. *gondii* infection exist already [4, 134, 253]. Animal models additionally allow for highly controlled study designs to assist initial delineation of mechanisms for a given sex or age. Large studies of clinical TBI populations should also be conducted in parallel to complement preclinical data. For example, *T*. *gondii* infection could be screened when TBI patients present to Emergency Departments, thus allowing for analyses of pathophysiological and outcome differences between TBI patients with and without *T*. *gondii*. Indeed, it is still not known how many latent *T*. *gondii* parasites are found in any tissue type in human let alone the brain. Current tests can only determine exposure by immunoglobulin G antibody response and are not informative about where latent forms reside in the human body. This is an important consideration and something that needs to be determined as we would only expect synergy of *T*. *gondii* and TBI if there were sufficient levels of parasites in the brain to exacerbate inflammation. Taken together, these studies are imperative in our understanding of how TBI and *T*. *gondii* interact, and provide a foundation to develop and optimize appropriate treatments for TBI patients with and without *T*. *gondii* to improve outcomes.

There are several existing drug candidates that could target overlapping mechanisms in *T*. *gondii* and TBI to minimize secondary injury. For example, the NLRP3 inhibitor MCC950, which has been demonstrated in several preclinical TBI studies to decrease neuroinflammation and improve cognitive and motor deficits, may prove efficacious when the conditions appear concomitantly [48, 49, 254]. MCC950 has additionally been demonstrated to decrease IL-1β secretion upon monocyte infection with *T*. *gondii* [255]; however, there is an absence of research pertaining to this inhibitor in in vivo chronic infection models. The IL-1 receptor antagonist, Anakinra, is another potential drug intervention in this context, and has already demonstrated safety and tolerability in human TBI trials [256]. Experimental studies have reported that Anakinra is beneficial in mice with a combined TBI and tibial fracture [257]. This injury combination results in exacerbated neuroinflammation compared to an isolated TBI [258], and may bear similarities to what would occur in a combined TBI and *T*. *gondii* setting.

The α2-adrenergic agonist Guanabenz may be another suitable drug candidate in the context of TBI combined with *T*. *gondii*, given its ability to downregulate inflammatory responses via elevation of eukaryotic initiation factor 2 alpha subunit (eIF2α) phosphorylation [259, 260]. Moreover, Guanabenz has been shown to be effective in reducing inflammation and cyst burden during the chronic stage of *T*. *gondii* [259, 261], as well as reduce endoplasmic reticulum (ER) stress and hence reduce neuronal loss post-TBI [262, 263]. Guanabenz may also be beneficial by decreasing sympathetic hyperactivity [264]. The eIF2α dephosphorylation inhibitor, Salubrinal, has additionally shown benefit in both *T*. *gondii* and TBI studies independently [265, 266]. In chronic *T*. *gondii* infection, Salubrinal has been demonstrated to inhibit the reactivation of bradyzoites [265]; and through TBI studies, it was established to suppress ER stress, as well as autophagy and apoptosis pathways, thereby reducing morphological and functional deficits post-injury [266]. Though Salubrinal does not target the overlapping neuroinflammatory pathways directly, it may still prove beneficial in reducing cell death acutely after injury, and bradyzoite reactivation if immunosuppression were to occur.

However, it is important to consider the possibility that these treatments may prove detrimental in those with a *T*. *gondii* infection, given that certain inflammatory processes are also necessary to control parasite replication. In other words, if an individual with a chronic *T*. *gondii* infection were to receive a neuroinflammatory-based drug candidate post-TBI, *T*. *gondii* tachyzoite replication may reactivate, resulting in uncontrollable parasite proliferation, exacerbated cell death, and clinical symptoms of toxoplasmosis. This therefore would have the opposite effect to what was intended. Hence, it would be beneficial to additionally investigate neuroinflammatory drug candidates with standard treatment strategies for reactivated toxoplasmosis, such as pyrimethamine-sulfadiazine therapy [267]. This particularly highlights the importance of future research investigating TBI coupled with *T*. *gondii* infection, in both the preclinical and clinical setting.

It would also be of interest to examine the incidence of TBI in individuals both with and without a pre-existing *T*. *gondii* infection. With growing evidence that *T*. *gondii* infection in and of itself may result in subtle behavioral abnormalities, it could be the case that these behaviors result in either increased or decreased risk of sustaining a future TBI. For example, *T*. *gondii* infection has been demonstrated to increase the risk of having a traffic accident [268]. As motor accidents are a common cause of TBI, these adults may consequently be at a higher risk of sustaining a TBI [18]. *T*. *gondii* seropositivity has also been associated with increased aggression and impulsivity in healthy adults [269]. These adults may engage in more risk-taking behavior than *T*. *gondii* seronegative individuals and additionally be at an increased risk of sustaining a TBI. Furthermore, this would align with a previous study demonstrating individuals with intermittent explosive disorder (which is characterized by impulsive aggression) were more likely to have a history of mild TBI [270].

As *T*. *gondii* seroprevalence increases with age, and those above 75 years of age account for a significant proportion of TBI-related hospitalizations and deaths, it would also be important to consider the incidence of TBI individuals with or without infection for this age group [120, 271]. Importantly, as aging is associated with immune system dysregulation as well as increased levels of basal inflammation, future studies investigating the effect of age on the hypothesized synergism of chronic *T*. *gondii* infection with TBI are essential [272].

Although the focus of this paper has been in the context of someone with chronic *T*. *gondii* experiencing a TBI, it should also be considered that a history of TBI may predispose individuals to worse outcomes upon a later *T*. *gondii* infection. For example, TBI can increase the proportion of activated microglia and neuroinflammation chronically after post-injury [55]. *T*. *gondii* would therefore be met upon migration to the brain parenchyma with a more robust neuroinflammatory response, potentially leading to excessive cell death. Moreover, if immunosuppression were to result from a TBI [79], and an individual were to be later infected with *T*. *gondii*, uncontrolled proliferation of tachyzoites may occur in enterocytes and once migrated into the brain parenchyma, exacerbated activation of apoptotic pathways may occur due to cell stress via tachyzoite proliferation, and necrotic tissue may result [273].

More broadly, it would also be of significance to determine whether peripheral parasitic infections, such as with enteric parasites, can alter TBI outcomes. Much like with a chronic *T*. *gondii* infection, a significant proportion of the clinical TBI population would be likely to encounter an intestinal parasite at some point either before or after injury. Amplified microglial activation, pro-inflammatory mediators, and functional deficits have previously been demonstrated through peripheral lipopolysaccharide challenge post-TBI [6, 274–276]. Additionally, as peripheral immunosuppression and brain-gut axis dysregulation can eventuate in the aftermath of a TBI, increased susceptibility to peripheral infection and increased mortality rate can occur [274, 276, 277]. Therefore, it is reasonable to predict a similar pattern of exacerbation would occur as a result of peripheral parasitic immunological stressors, among other types of peripheral infections. However, to date, this has been an understudied topic, and this field of research requires greater attention in future studies.

By the same token, it is important to consider how other common neurologically involved parasitic infections may alter TBI pathophysiology and outcomes. For example, *Plasmodium falciparum* has been attributed to upward of half a million deaths annually, and can result in cerebral malaria [278, 279]. Cytoadherence of parasitized erythrocytes to the endothelium can initiate inflammatory pathways and may contribute to BBB disruption [279]. Therefore, coupled with the BBB alterations that are commonplace post-TBI, an exacerbated pro-inflammatory response may occur alongside excessive neurovascular damage and increased edema. As cerebral malaria is a risk factor for epilepsy, it would also be of importance to investigate whether a *Plasmodium* infection paired with a TBI could increase the incidence of PTE [280, 281]. Additionally, members of the *Trypanosomatidae* family warrant investigation as potential modifiers of TBI outcomes, particularly given that human African trypanosomiasis presents with a late meningoencephalitic stage [282]. African trypanosomes are capable of crossing the BBB into the brain parenchyma and increased expression of pro-inflammatory mediators, such as TNFα, IFNγ, and CXCL10, is believed to assist parasitic invasion of the CNS [283, 284]. However, the signaling pathways involved have not been extensively studied. As circadian rhythm disturbances are commonly reported post-TBI, another intriguing prospect for interplay between African trypanosomes and TBI would be in regard to circadian rhythm alterations given that African trypanosomes are notoriously known to reverse sleep cycles [282, 285]. Chagas disease, which is also known as American trypanosomiasis, is common in Latin American stroke patients and can present with highly varied neurological manifestations [286, 287]. However, as cardiac and digestive tissues are predominantly infected, with cardiomyopathy being the main clinical feature in the chronic stage, neuroinflammatory events have not been well characterized for this disease and the capacity to speculate about a potential overlap with TBI pathophysiology is limited [288]. Ultimately, the high prevalence and unique neuropathology of a *T*. *gondii* infection, coupled with the shear lack of etiological knowledge surrounding cerebral complications of malaria and trypanosomiasis, further solidifies *T*. *gondii* as the prime candidate to begin investigations between parasitic infection and TBI.

## Conclusions

In closing, TBI is a key contributor to the global burden of disease but despite promising preclinical trials, to date no effective treatments exist. This reflects the heterogeneity of TBI pathophysiology and presentation, such as the presence of infection. *T*. *gondii* chronically infects approximately one-third of the world’s population, which equates to a significant proportion of individuals who sustain a TBI being chronically infected with *T*. *gondii* at the time of insult. As there are a myriad of neuroinflammatory processes that are common to both conditions, exacerbated neuroinflammation, amplified functional deficits, and accelerated neurodegenerative processes may occur in TBI individuals who are confirmed with chronic *T*. *gondii* infection. The interplay between *T*. *gondii* and TBI however remains speculative and as such, further investigations should be conducted to assist TBI treatment development and future clinical practice.

## Data Availability

Not applicable.

## Abbreviations

AAH: Aromatic amino acid hydroxylase
AD: Alzheimer’s disease
ATP: Adenosine triphosphate
BBB: Blood-brain barrier
CCL: CC chemokine ligand
CNS: Central nervous system
CXCL: CXC chemokine ligand
DC: Dendritic cell
eIF2α: Eukaryotic initiation factor 2 alpha subunit
ER: Endoplasmic reticulum
GABA: Gamma-amino butyric acid
GLT: Glutamate
GM-CSF: Granulocyte-macrophage colony-stimulating factor
HMGB1: High-mobility group box 1
HPA: Hypothalamic-pituitary-adrenal
IL: Interleukin
IFNγ: Interferon-γ
MyD88: Myeloid differentiation primary response protein 88
NF-κB: Nuclear factor κ-light-chain-enhancer of activated B cells
NLRP3: Nucleotide-binding oligomerization domain-like receptor family pyrin domain-containing 3
NO: Nitric oxide
PRR: Pattern recognition receptor
PTE: Post-traumatic epilepsy
ROS: Reactive oxygen species
SNS: Sympathetic nervous system
TBI: Traumatic brain injury
TLR: Toll-like receptor
TNFα: Tumor necrosis factor-α
